# Network machine learning maps phytochemically rich “Hyperfoods” to fight COVID-19

**DOI:** 10.1186/s40246-020-00297-x

**Published:** 2021-01-02

**Authors:** Ivan Laponogov, Guadalupe Gonzalez, Madelen Shepherd, Ahad Qureshi, Dennis Veselkov, Georgia Charkoftaki, Vasilis Vasiliou, Jozef Youssef, Reza Mirnezami, Michael Bronstein, Kirill Veselkov

**Affiliations:** 1 Department of Surgery and Cancer, Faculty of Medicine, Imperial College, London, SW7 2AZ UK; 2 Department of Computing, Faculty of Engineering, Imperial College, London, SW7 2AZ UK; 3 Intelligify Limited, 160 Kemp House, City Road, London, EC1V 2NX UK; 4 Department of Environmental Health Sciences, Yale School of Public Health, New Haven, CT USA; 5 Kitchen Theory, London, EN5 4LG UK; 6 Department of Colorectal Surgery, Royal Free Hospital, Hampstead, London, NW3 2QG UK; 7 Twitter, 20 Air St, London, W1B 5DL UK; 8 Faculty of Informatics, University of Lugano, Via Giuseppe Buffi 13, Lugano, 6900 Switzerland

**Keywords:** Machine learning, Antiviral, COVID-19, SARS-CoV-2, Drug repositioning, Food, Interactomics, Gene-gene networks

## Abstract

In this paper, we introduce a network machine learning method to identify potential bioactive anti-COVID-19 molecules in foods based on their capacity to target the SARS-CoV-2-host gene-gene (protein-protein) interactome. Our analyses were performed using a supercomputing DreamLab App platform, harnessing the idle computational power of thousands of smartphones. Machine learning models were initially calibrated by demonstrating that the proposed method can predict anti-COVID-19 candidates among experimental and clinically approved drugs (5658 in total) targeting COVID-19 interactomics with the balanced classification accuracy of 80–85% in 5-fold cross-validated settings. This identified the most promising drug candidates that can be potentially “repurposed” against COVID-19 including common drugs used to combat cardiovascular and metabolic disorders, such as simvastatin, atorvastatin and metformin. A database of 7694 bioactive food-based molecules was run through the calibrated machine learning algorithm, which identified 52 biologically active molecules, from varied chemical classes, including flavonoids, terpenoids, coumarins and indoles predicted to target SARS-CoV-2-host interactome networks. This in turn was used to construct a “food map” with the theoretical anti-COVID-19 potential of each ingredient estimated based on the diversity and relative levels of candidate compounds with antiviral properties. We expect this in silico predicted food map to play an important role in future clinical studies of precision nutrition interventions against COVID-19 and other viral diseases.

**Supplementary Information:**

The online version contains supplementary material available at 10.1186/s40246-020-00297-x.

## Background

The rapid and continued spread of severe acute respiratory syndrome coronavirus 2 (SARS-CoV-2) is resulting in persistent outbreaks of novel coronavirus disease 2019 (COVID-19) across the world [1]. This in turn is having damaging effects on global economies and healthcare systems, wellbeing, mental health and societal dynamics, as a whole. In the absence of effective curative treatments and validated vaccines, there is an urgent need for innovative solutions. Combining conventional medical treatments with nutritional interventions represents one such solution, which is gaining traction [2, 3]. Considerable recent efforts have been directed towards identifying new purposes, or alternative uses, for existing drugs (so-called drug repurposing) [4, 5]. This offers an attractive way to circumvent the slow and costly pathway to new drug development and regulatory approval. Several examples of repurposed drugs have been tested or are currently being tested in clinical trials for deployment against COVID-19 [6]. In particular, the randomised controlled trials of the corticosteroid dexamethasone have confirmed its capacity to reduce mortality by up to a third in COVID-19 patients admitted to hospital for respiratory support [7]. However, there are no clinically approved drugs or other antiviral therapeutics for COVID-19 prevention, or for the treatment of non-hospitalised symptomatic patients. These patients are typically discharged home with basic advice, but remain at risk of personal clinical deterioration (especially those with underlying comorbidities) and also pose an ongoing risk to close contacts.

The human diet is rich with molecules that have been shown to play a role in both the prevention and treatment of viral diseases, by interacting with drugs to enhance their potency or by acting as “medicines” themselves [8]. Of particular relevance are plant-based foods which possess a complex profile of molecules of varied chemical classes such as alkaloids, flavonoids, coumarins, terpenoids and indoles [9]. Laboratory studies have revealed multiple mechanisms of action by which these dietary compounds exert their action against functionally and genetically diverse viruses [10, 11]. Furthermore, there is a growing body of evidence that poor dietary habits and diet-related comorbidities such as obesity, diabetes and cardiovascular disease are at least partially responsible for disparities in adverse outcomes from COVID-19 across the globe [12, 13]. One possible explanation for this could be poor gut microbiome health and pre-existing pro-inflammatory state leading to a dysregulated cytokine storm among vulnerable COVID-19 patients that is associated with the high mortality of such cases [14].

Identification of dietary constituents and consequent design of phytochemically rich “Hyperfoods” with disease-beating properties can be a safe and cost-effective method for developing tailored nutrition-based therapeutic strategies against many diseases, including COVID-19 [15]. However, it is vitally important to appreciate that the modern era of molecular gastronomy has resulted in a growing expectation for food to fulfil taste, aesthetic, sensory and health-centred requirements. For these reasons, the design of such “Hyperfoods” requires multi-faceted optimisation, taking into account not only pro-health benefits but also considering visual aesthetics (e.g. colour, texture) and sensory (e.g. taste mouthfeel) characteristics [15]. At present, the landscape of potential drug-like molecules in food is unimaginably vast. Thanks to advances in high-throughput mass spectrometry technologies and machine learning, identification and molecular networking of thousands of these molecules from various food sources has become possible [16]. Investigating the influence of a single drug or food component on any particular viral infection takes months to years of experimental research. Examples of experimentally derived phytochemicals with antiviral properties include hesperedin and naringin in citrus foods, tannic acid in black tea, emodin in rhubarb and myristicin in dill and parsley [17, 18]. Given the vast molecular space, the traditional practicalities of investigating the influence of a single molecule or food component would take far too long to have an impact on the current COVID-19 crisis.

Coronaviruses cannot survive or replicate without host assistance. In fact, all viruses have naturally evolved a sophisticated array of molecular strategies designed to exploit the host’s cellular machinery to promote viral survival and replication. These strategies rely on a complex network of physical interactions between viral and host genes and proteins (so-called virus-host interactome networks, here and further due to the specifics of the existing interaction datasets, “gene” and “protein” terms can be used interchangeably) [19]. The conventional antiviral drug development paradigm assumes that one drug targets one viral protein [20]. In this regard, molecular docking computational simulations have been extensively performed to discover plant-based bioactive molecules for specific SARS-CoV-2 protein targets [21]. This approach has multiple drawbacks among which is the robustness of complex virus-host interaction networks to individual protein perturbations. The putative effects of vaccines and drugs against SARS-CoV-2-specific gene or protein targets can also be complicated by escaped viral mutants [22].

Here, we hypothesise that an effective anti-COVID-19 preventative or therapeutic intervention should target multiple biochemical networks implicated in virus entry and pathogenesis such as angiotensin-converting enzyme-2 (ACE2)/G protein Mas receptor (MasR) axis, mitogen-activated protein kinase (MAPK) cascade, and toll-like receptor signalling pathways [23]. Building on our previous work on cancer-beating molecules from food sources [15] and other recent network medicine studies for computational drug repurposing against COVID-19 [24], we have combined network-based machine learning methods, mobile supercomputing and interactomics data to identify food-based bioactive molecules targeting SARS-CoV-2-human interactome networks. We have first calibrated the proposed machine learning workflows to predict experimentally confirmed drugs with anti-COVID-19 properties. Once calibrated, the models were used to discover drug-like molecules in foods. The discovered molecules/sources were used to compile a list of antiviral “Hyperfoods” weighted by the highest diversity and levels of antiviral molecules against SARS-CoV-2-human interactome networks. We envision that the list of phytochemically rich “Hyperfoods” revealed in this work will serve as a fundamental pillar in the design of a precision nutrition intervention strategy against COVID-19.

## Results and discussion

### Genome-wide network-based machine learning for predicting drug and food molecules targeting SARS-CoV-2-host interactome

We have used the random walk propagation algorithm to learn the effects of SARS-CoV-2 on human interactome networks governing regulatory and biochemical pathways. The SARS-CoV-2 virus exploits human biomolecular network machinery to promote viral entry, survival, replication, spread and shedding. The propagated SARS-CoV-2-host interactome profile was subjected to the Gene Set Enrichment Analysis (GSEA), which highlighted multiple potential mechanisms by which the coronavirus exerts its activity on the host (Additional file 1). These include membrane surface proteins (ACE2), regulation of programmed cell death pathways (caspase 8 and p38/MAPK signalling), genomic replication pathways (RNA polymerase pathways), immune-modulatory signalling circuits (toll-like receptors, the nuclear factor-kB (NF-kB), JAK/STAT signalling pathways) and inflammatory axes (e.g. interleukin pathways; see section 4 of Additional file 2 for additional details).

**Fig. 1:**
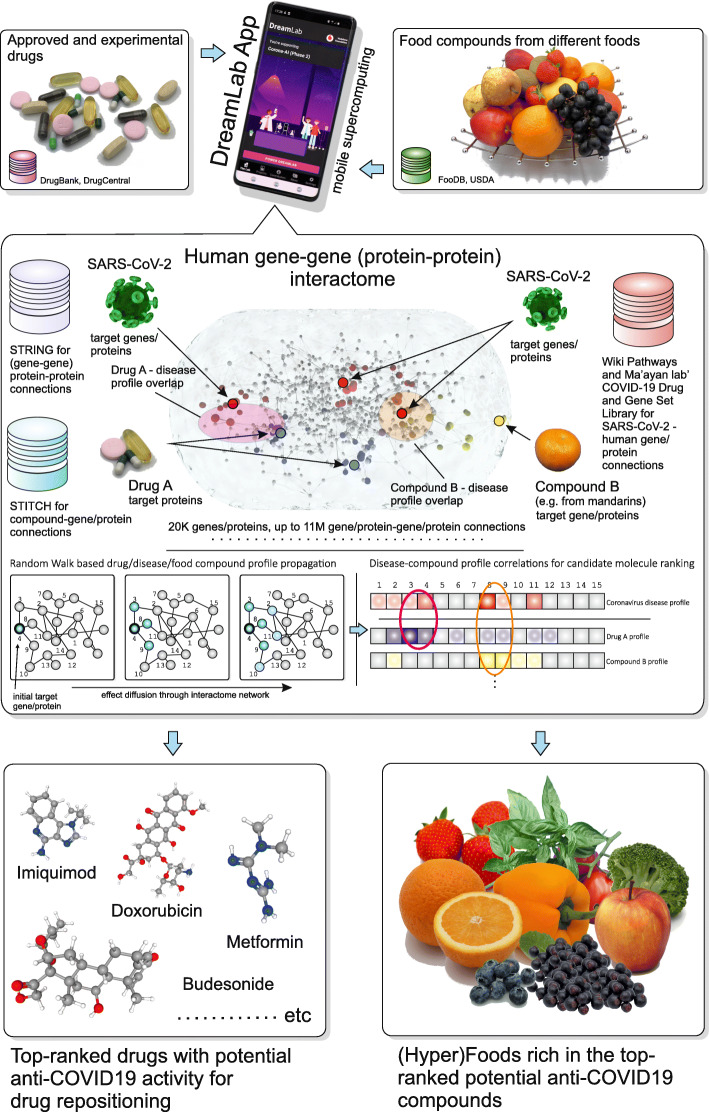
Schematic diagram of the overall workflow. The random walk with restarts algorithm operating within a mobile supercomputing DreamLab App is used to simulate how drug and food-based compounds interact with COVID-19-associated viral gene/protein networks. This has been extrapolated from human genome-wide gene-gene (protein-protein) interactome data and based on known COVID-19 human proteome viral targets (i.e. human genes/proteins interacting with different stages of the virus life cycle to facilitate replication and/or enhance viral potency). Both disease and molecular compound impacts are propagated through the interactome network to model the overall cellular response/interactome perturbation. The resulting compound and disease profiles are then correlated to rank compounds according to their network “overlap” with “reference” viral profiles. This approach is based on the assumption that to have an effect, candidate compounds should target the same network component(s) as the one(s) disrupted by the virus. Therapeutic effect can be direct, or indirect, for example where compounds are found to interact with neighbouring network nodes, resulting in subsequent effect propagation to the desired target

The ranking of drug and food molecules was based on their potential interaction capability with COVID-19, which in turn has been derived from their respective effects on the human protein-protein (or gene-gene) interaction network, commonly referred to as the interactome. The main assumption here is that for a given molecule to have an effect against coronavirus, it should target the same pathways and cellular mechanisms targeted by the disease, but with the opposite regulatory effect. This action does not necessarily imply that gene/protein targets have a direct effect, and the effect can be indirectly exerted through other neighbouring proteins in the network, via gene-gene (protein-protein) interaction. This approach permits modelling the systemic genome-wide response to the disease and drug/food intervention and identifying drug/food-based compounds with the highest probability of being effective against COVID-19 (see Fig. 1). Similar network propagation approaches have been applied in cancer research for drug repurposing [24], for mutation-driven population stratification [25] and, in our earlier work, for drug repurposing and food-based anti-cancer molecular therapeutics [15]. Although there are other approaches being developed for drug repurposing using multi-omics and phenotypic data [26], these mandate additional datasets that are usually not available for food-based molecules.

The machine learning algorithm parameters were calibrated for predicting experimentally validated drugs against COVID-19 in a cross-validation setting (see section 3 in Additional file 2). The optimal balanced classification accuracy in the range of 80–84.9% was achieved using an ensemble of parameter settings (3609 models for aggregated interactome (see Additional file 3) and 15 models for manually curated interactome derived from a biological pathway database of COVID-19 WikiPathways (see Additional file 4)). Practically, this resulted in approximately 8 out of 10 drugs being correctly classified into their respective classes (i.e. potentially anti-COVID-19 vs others). For each parameter combination achieving balanced accuracy above 80%, a ranked list of compounds (drugs and food molecules) was generated with compounds ranked by the decreasing correlation between compound and disease profiles. The consensus list of top-ranked compounds with the highest antiviral ranking and probability is summarised in Additional file 5. For each candidate molecule, we also provided a putative mechanism of action and literature reference where available.

### Drug repositioning candidates against COVID-19

Our analysis identified imiquimod as the top-ranked drug with anti-COVID-19 potential. Imiquimod acts as an agonist of toll-like receptor 7, which is crucial in recognising single-stranded RNA viruses, such as SARS-CoV-2. Toll-like receptors generate antiviral immunity and act to induce favourable type I interferon response, which in turn induces the expression of interferon-stimulated genes leading to the inhibition of viral replication [27].

Several widely used chemotherapeutic agents were found to exert potential anti-COVID-19 effect, including doxorubicin, fluorouracil and gemcitabine. Doxorubicin is commonly used in the treatment of advanced breast cancer, bladder cancer and lymphoma, as well as a number of other malignancies. A previous study has indicated that SARS-CoV-2 contains residues that are vulnerable to the reactive glycating agent methylglyoxal, cellular levels of which are increased by doxorubicin [28]. Fluorouracil is a fluoropyrimidine used for the treatment of a number of solid organ tumours. It is a precursor of deoxythymidine triphosphate and uridine-5′-triphosphate (UTP) during biogenesis and interferes with both DNA and RNA metabolism. This drug is preferentially incorporated into RNA instead of UTP, which interferes with RNA processing and protein synthesis and this in turn can lead to the disruption of viral RNA replication and elicit an antiviral effect [29]. Gemcitabine has also been shown to inhibit SARS-CoV-2 replication. It is hypothesised that this effect occurs through targeting of pyrimidine biosynthesis salvage pathways and stimulation of the innate immune system [30]. Although chemotherapy and other anti-cancer treatments may result in significant immune compromise in patients, rendering them more susceptible to viral and other infectious illnesses [31], the findings presented here also highlight a *double-edged* phenomenon, whereby they may actually exert potential beneficial effects against COVID-19 infection.

Statins are considered a clinically important breakthrough in the prevention and treatment of cardiovascular disease. Simvastatin and atorvastatin were found to offer significant anti-COVID-19 potential. The hypothesis is that statins in general reduce COVID-19 infectivity through the removal of cholesterol used by SARS-CoV-2 to infect cells [32] and reduce the risk of cardiovascular complications that are symptomatic of severe COVID-19 infection. In addition, they may enhance innate immune responses to viral infections through inhibition of the myeloid differentiation primary response 88 signalling pathway. Correspondingly, a recent meta-analysis of data from multiple studies reported a 30% reduction in fatal or severe disease course in patients with confirmed COVID-19 infection who were taking statins [33].

Metformin is globally regarded as one of the key pharmacotherapies in the management of diabetes mellitus. Of note, it was originally introduced as an anti-influenza drug, with glucose-lowering capability regarded as a side-effect of treatment, rather than desired primary endpoint. The many pleiotropic effects of metformin together with its widespread utility in modern medicine have earned it the name “the aspirin of the 21st century” [34]. It activates the AMP-activated protein kinase, resulting in the phosphorylation of angiotensin-converting enzyme II (ACE2), which leads to conformational and functional changes to ACE2 that are thought to inhibit SARS-CoV-2 binding and/or entry [35]. In support of these suggestions, a recent meta-analysis demonstrated a reduced risk of mortality in COVID-19 patients receiving metformin [36].

### Prediction of “dark matter” of food biochemistry with anti-COVID-19 properties

**Fig. 2:**
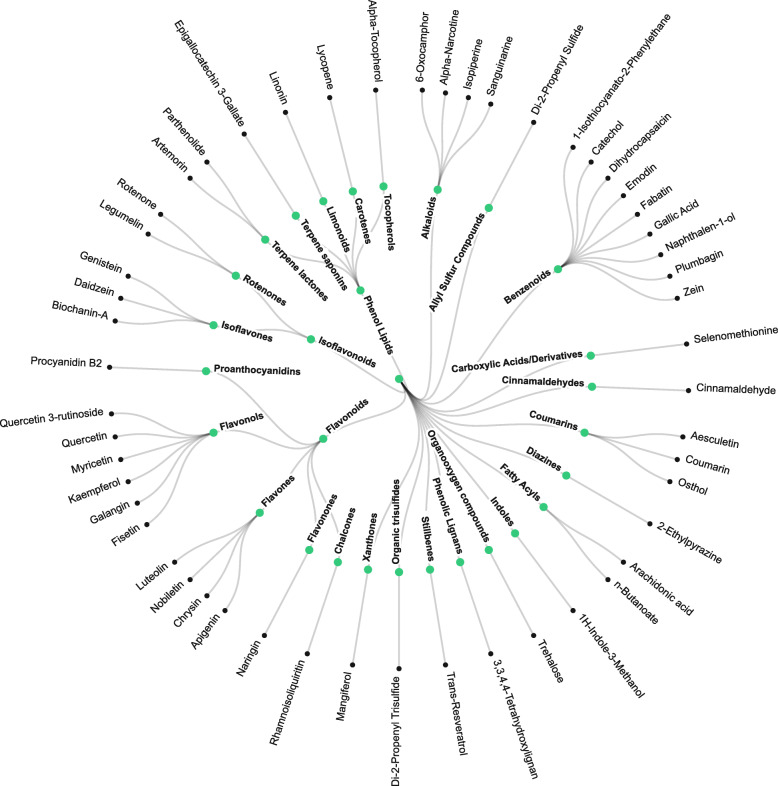
Hierarchical classification of the top 52 predicted antiviral molecules targeting SARS-CoV-2 human interactome networks

In addition to minerals, vitamins and micronutrients, all plant-based foods contain phytochemicals that are non-nutritive components in the diet but can exert protective or disease-beating effects. This phytochemistry has been exploited extensively for the development of antiviral drugs with more acceptable side-effect profiles, compared to synthetically generated drugs [37]. The network-based analysis presented here identified 52 food-based molecules based on their capability to target SARS-CoV-2-host interactomes. These molecules belong to a variety of chemical classes including (iso)flavonoids, terpenoids, phenols and indoles (see Fig. 2). As highlighted, the presence and abundance of these molecules are not typically monitored by national nutritional agencies, which conventionally focus on minerals, vitamins and macronutrients. These compounds can be regarded as the “dark matter” of nutritional science. Because of their bitter taste, it is interesting to note that the food industry routinely removes some of these compounds through selective breeding and a variety of debittering processes to improve taste [38]. This has even led to the suggestion by some cancer research groups that foods possessing more bitter taste may actually offer greater health benefits [38].

The (poly)phenolic classes of molecules such as flavonoids, coumarins, stilbenes, indoles and phenolic acids make up the majority of anti-COVID-19 bioactive compounds identified by our network-based machine learning algorithm. These include flavonols (e.g. quercetin, kaempferol and myricetin), flavones (e.g. luteolin and apigenin), flavanols (e.g. procyanidin B2), flavanones (naringin), isoflavonoids (daidzein, genistein and legumelin) as well as stilbenes (trans-resveratrol), indoles (3-indole-carbinol) and phenolic acids (gallic acid). In edible plants such as fruits and vegetables, phenolic molecules are widespread and contribute to their aroma, taste and colour. These compounds are synthesised in abundance by plants in response to environmental stimuli and play an indispensable role in defence against pathogens (including viruses) and insects [39]. Their ability to disrupt the life cycle of SARS-CoV-2 is partially achieved via interference with viral proteins. For example, among our top-ranked molecules, epigallocatechin 3-gallate was demonstrated experimentally to inhibit 3-chymotrypsin-like protease (3CLpro) [38]; quercetin demonstrated binding affinity to inhibit 3CLpro and papain-like protease (PLpro) [40], while trans-resveratrol inhibits nucleocapsid (N) proteins [18].

In addition, the identified compounds appear to mitigate against various patho-physiological processes that develop in response to COVID-19. For example, regulation of the renin-angiotensin system (RAS) and expression of angiotensin-converting enzyme 2 (ACE2), stimulation of immune system, downregulation of pro-inflammatory cytokine release and amplification of cytotoxic T lymphocyte (CTLs) and natural killer (NK) immune cell pools. The putative mechanism of action for each of the identified compounds is summarised in Additional file 5.

### Construction of anti-COVID-19 food map

The potential for particular foods to exert COVID-19 preventative and/or therapeutic effect depends upon the bioavailability and diversity of bioactive molecules with antiviral properties contained therein [41]. A key limitation of the existing literature on food-based compounds is the largely over-simplified view that is commonly taken, whereby studies have tended to focus on specific molecular components in isolation, for example specific flavonoids such as quercetin [42]. However, when candidate antiviral agents acting in isolation have been evaluated in clinical studies, they have failed to consistently confer the same level of benefit [43]. It seems more plausible that consumption of whole foods, with their associated phytochemicals *en masse* may provide greater health benefits, due to molecular additive or synergistic effects. It therefore follows that the antiviral properties of a given food will be governed by two key factors: (1) the additive, antagonistic and synergistic actions of their individual components and (2) the way in which these simultaneously modulate different intracellular pathways involved in SARS-CoV-2 pathogenesis.

**Fig. 3:**
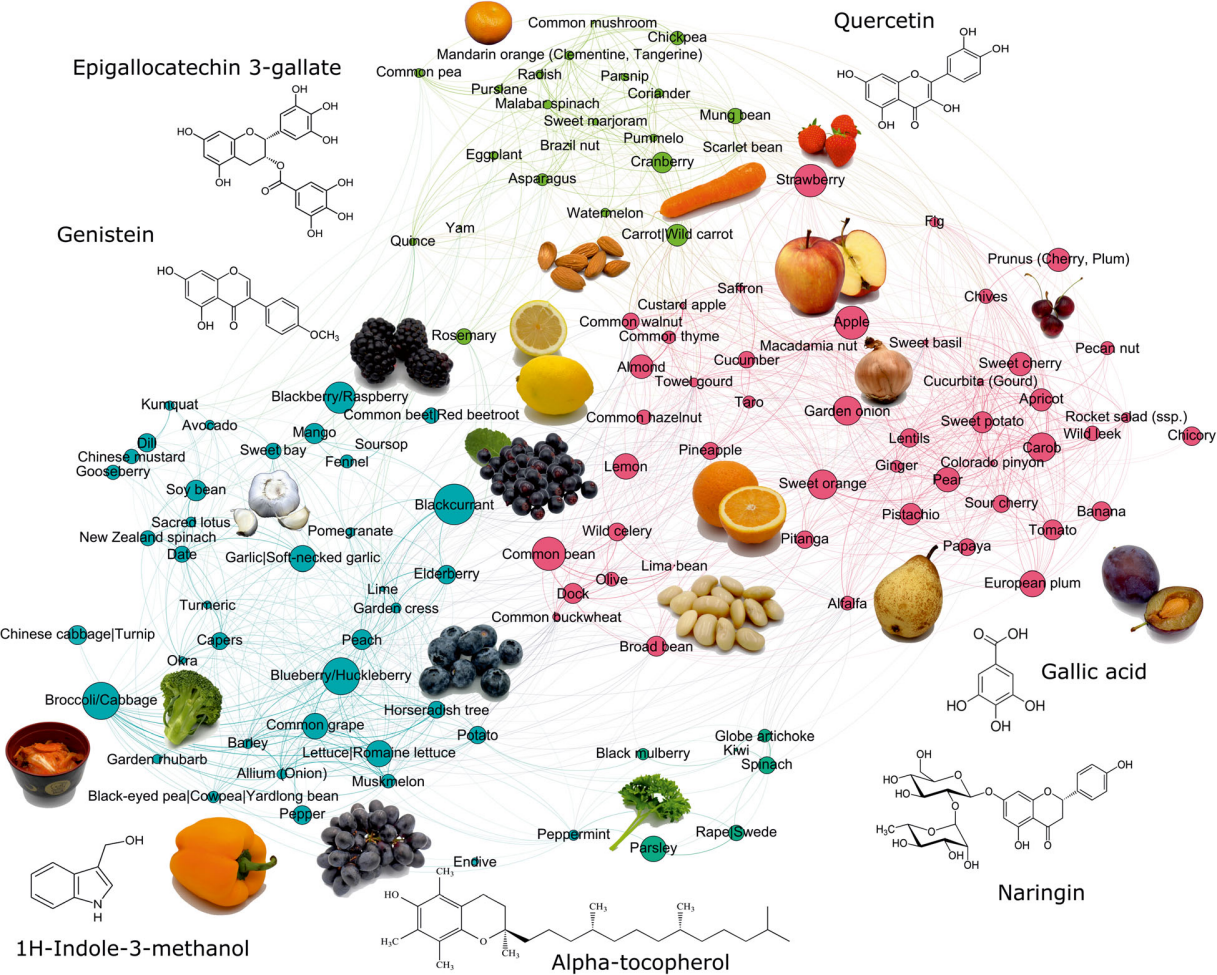
The contained profiles of compounds within specific foods, with predicted effectiveness in targeting SARS-CoV-2-host interactome networks. Each node in the figure denotes a particular food item, and node size in each case is scaled by the derived enrichment score based on the diversity and relative levels of molecules with predicted anti-COVID-19 properties. The links between nodes reflect the pairwise correlation (“similarity”) antiviral profiles in foods; thus, the clusters of foods illustrate molecular commonality between them. A list of foods, constituent food compounds and enrichment scores can be found in Additional file 8

Based on these assumptions, we have constructed a food map with the theoretical anti-COVID-19 capacity of each ingredient ranked according to an “enrichment score” derived from the diversity and relative levels of candidate compounds with antiviral properties (Fig. 3; see section 5 of Additional file 2 for more details). To identify putative mechanisms responsible for the anti-COVID-19 properties of predicted foods, we have simulated the effects of a phytochemical profile of a given food item on human interactome pathways and sub-networks, using the random walker algorithm and gene set enrichment analysis. The analysis showed that the most influential impacted pathways by predicted phytochemically enriched foods with anti-COVID-19 properties exhibited a statistically significant overlap with SARS-CoV-2 disrupted pathways (Additional file 6). This implies that a phytochemical profile of food ingredients, rather than individual molecules, exert a combined effect across multiple host pathways affected by SARS-CoV-2 (see section 4 of Additional file 2 for additional details).

The top-ranked phytochemically rich food sources (called “Antiviral Hyperfoods”) include different berries (blackcurrant, cranberry and blueberry), cruciferous vegetables (cabbage, broccoli), apples, citrus fruits (sweet orange and lemon), onions, garlic and beans. A recent study highlighted the potential of cabbage and fermented vegetable consumption in minimising adverse outcomes in COVID-19, supporting our results [44]. The present analysis has demonstrated that this is potentially due to a profile of anti-COVID-19 compounds from various molecular classes rather than individual molecules as previously suggested (see Additional file 6). Similarly, the complex antiviral molecular profile of berries such as blackcurrant and blueberries may explain their experimentally observed potency against genetically and phenotypically diverse viruses [45], though their ability to protect specifically against COVID-19 is yet to be evaluated in clinical trials.

## Conclusions

Current methods for prevention, treatment and containment of COVID-19 have not been effective in curbing the rate of transmission. Figures across the world show a sustained rise in cases. Non-hospitalised patients are discharged home where they continue to pose a risk to close contacts, and where they are at ongoing risk of clinical deterioration (especially those with comorbidities such as diabetes, obesity and cardiovascular diseases). For this group of patients, there is a critical need for innovative and cost-effective out-of-hospital treatment. The use of precision nutrition strategies is safe and highly promising in this context. Using a network-based machine learning method, we have shown that certain plant-based foods such as berries, cruciferous vegetables, apples, citrus fruits, onions, garlic and beans are most enriched in terms of the diversity and relative abundance of bioactive molecules targeting the SARS-CoV-2-human interactome.

We acknowledge that the present work is subject to a number of limitations. Firstly, the cultivation, storage and cooking methods may influence bioactive molecular composition in foods. Secondly, it remains unclear whether these compounds would be present in sufficient levels to exert beneficial biological activity. Thirdly, the identified phenolic compounds can be filtered out by food producers because of their bitter taste to enhance palatability and taste experience. This raises interesting practical issues for “Hyperfoods” because increasing the content of bitter phytonutrients for health benefits may not be entirely compatible with consumer acceptance. Fourthly, the proposed methodology only accounts for interactions between bioactive food compounds and SARS-CoV-2-human-related molecular networks, without necessarily defining the directionality of these relationships. Fifthly, the methods described here do not take into account specific COVID-19 individual molecular phenotypic characteristics. Finally, drug combinations, drug-drug and drug-food interactions have not been evaluated in this study; as such, it is not clear whether these will lead to synergistic or antagonistic effects where they act on common molecular networks, or whether this combination will disrupt drug metabolism itself. This will be the subject of the investigation for future phases of the CORONA-AI project. Nevertheless, these considerations notwithstanding, we expect this in silico predicted food map to play an important role in future clinical studies of precision nutrition interventions against COVID-19. In the near future, the goal will be to develop a personalised “food passport” for each patient, designed to provide “smarter” food choices with the ability to reduce susceptibility to COVID-19 infection and mitigate against severe and long forms of the disease. Further clinical validations of our findings are needed in a randomised double-blind placebo-controlled trial setting.

## Methods

### Corona-AI/DreamLab mobile cloud supercomputing

The results presented in this manuscript were derived from the Corona-AI: phase I project for interactome-driven drug and food compound search for the potential anti-COVID-19 treatment. Working with Vodafone Foundation, the “Corona-AI” project used the freely available DreamLab app, which runs calculations using a smartphone computing power while its user sleeps. Tens to hundreds of thousands smartphones combined together are used to crunch scientific data at scale rivalling available supercomputers and by far exceeding the capabilities of the normal desktop PCs. The DreamLab App can be freely downloaded by anyone willing to donate the unused computational power of their smartphone to cancer and coronavirus research. The DreamLab App runs when a smartphone is being charged: it loads a small portion of scientific data from the cloud, performs the computations and sends the results back to the cloud for scientists to analyse them. This way anyone can become a citizen scientist and contribute to global research.

### Learning propagation profiles of drugs, foods and diseases

The main assumption of the methodology used in this paper was that the drugs/food molecules which were effective at treatment of the particular disease would have a similar pattern of affected genes/proteins to the pattern of the genes/proteins affected by the disease. Due to gene-gene (or protein-protein) interactions within the cell, disease and drug do not necessarily have to affect exactly the same genes/proteins—their effects can be exerted on different, but interlinked proteins and propagated through protein-protein networks. For that, the aggregated 20,256 genes/proteins were represented as an array of floats where each value represents how strongly the protein was affected/perturbed by the disease or the drug (further referred to as drug or disease profiles). Zero value would mean no effect or a normal unperturbed state.

Gene-gene (protein-protein) connections and drug-gene/protein connections were filtered according to their confidence level (from STRING and STITCH databases) before disease and drug profile generation and propagation (these thresholds were among the adjustable parameters). Optionally, the number of drug-gene/protein connections was also capped at specific value equalising compounds with vastly different numbers of known connections. If this option was used, the top N connections are taken for each compound and the minimal connection score is established from them. Then the connections are thresholded according to this minimal connection score. This allowed one to include connections with the same score as the last one in top N.

Genes/proteins directly affected by the drug or disease receive the initial value of 1.0 (or the score weight in case of “score_5_weighted” target protein selection). Then the array of gene/protein perturbations was normalised to the sum of 1.0.

Random walk algorithm with restarts was used to propagate the perturbation of the genes/proteins through the network. For a detailed definition of the algorithm, we refer the reader to section 1 of Additional File 2.

The random walk with restarts was applied to simulate the perturbations of direct virus-host protein targets on the whole human interactome. It transforms a short list of genes/proteins directly targeted by the virus into a genome-wide profile of gene scores based on their network proximity to target candidates (referred as the “SARS-CoV-2 genome-wide response profile”). The same random walker algorithm was then used to get the activity profiles of candidate molecules, i.e. drugs or food-based compounds. The Pearson correlation coefficient between propagation profiles of food/drug compounds and COVID-19 disease was used to rank compounds that target SARS-CoV-2-host interactome networks. The formulation of the Pearson correlation coefficient can be found in section 2 of Additional file 2.

The parameter settings for interactomes and diffusion processes for compound ranking were optimised as described in section 3 of Additional file 2

#### Compound-protein and protein-protein interactome construction

The interactome used in this study was constructed as was described previously in [15]. In brief, a human genome was constructed from gene or protein sequences from COSMIC [46], NCBI Gene [47], STRING [48] and UniProt [49] databases. Fifteen thousand nine hundred eleven protein sequences matched exactly between databases, 1532 protein sequences were matched as subsections of larger sequences and 1686 proteins were matched allowing up to 5% amino acid mismatch. One thousand one hundred twenty-seven mismatched sequences were also included in the final unified set of 20,256 gene-encoded proteins. The list of genes/proteins (these two terms are used interchangeably with regard to the interactome analysis) was further populated with different gene IDs and synonyms including Ensembl and HGNC. Protein-protein interactions were obtained from STRING (~ 11M connections) and BioPlex (~ 100K connections) databases [50] and supplemented with confidence scores (0–999) from STRING.

Drug-protein interactions were obtained from the STITCH database [51], scored by the confidence level of 0–999 for drugs from DrugBank [52] and DrugCentral [53] databases as well as food molecules from FooDB [www.foodb.ca]. Indications for drugs and FDA approval status were extracted from DrugCentral.

#### Coronavirus target protein aggregation

Two recent sources for the coronavirus-affected sets of human genes/proteins were used in this work:

1. The COVID-19 Drug and Gene Set Library which provides a collection of drug and gene sets related to COVID-19 research aggregated from multiple sources using natural language processing techniques (downloaded on 24 September 2020) [54]. This set of genes/proteins is further referred to as the “Aggregated” set. In this set, human genes/proteins are scored by the number of times they have been reported as related to COVID-19 with the top score of 88 assigned to STAT1 gene. We generated several subselections of genes with different cut-offs for the scores: 40, 30, 25 and 20 (counting 72, 143, 248 and 457 genes) referred to as “score_40”, “score_30”, “score_25” and “score_20” respectively. In these subselections, the genes are all initially equally weighted when propagated through interactome and the chosen score threshold serves as an adjustable model parameter. We also included a set of 5000 top genes (with a minimal score of 5, referred to as “score_5_weighted”) with each gene weighted by its score for the propagation and a minimal entry set (consisting of CTSB, CTSL, TMPRSS2 and ACE2 genes [55], first reported in the literature as involved in the initial entry of the virus) referred to as “entry_only”.
2. COVID-19 Pathways Portal on WikiPathways [23] was used to create a subset of 423 coronavirus-affected human genes/proteins referred to as “score_wiki”.

#### Compound selection

SARS-CoV-2 is a relatively new pathogen, and there is a very limited number of experimentally validated drugs which were shown to be effective against it. We manually curated a list of such compounds from a literature search (see Additional file 7). Special care was taken to include only compounds with experimental rather than predicted evidence and explainable mechanism of action. Preference was given to the compounds already included in clinical trials. This resulted in a list of 49 “positive” class compounds. Drugs were putatively classified into two subgroups—acting through cellular mechanisms directly against the virus (“Direct-Cell”) or having symptomatic effects, e.g. anti-inflammatory (“Symptomatic”). This classification is not strict as drugs may have overlapping functions. Finally, drugs were checked for direct target overlaps with the “Aggregated” set of COVID-19-related genes. Drugs with very few to no overlaps in the top 100 genes were marked as less “reliable”. This resulted in four subselections for the “positive” class compounds which were tried in the model parameter optimisation stage: (1) “Target_Cell”: all drugs acting directly on the host-viral interactome, 27 in total; (2) “Target_Cell_Strict”: same as above, but only the most “reliable” drugs included, 19 in total; (3) “Target_Cell_Sympt”: both symptomatic and host-viral interactome targeting drugs included, 49 in total; and (4) “Target_Cell_Sympt_Strict”: same as above, but only the most “reliable” drugs included, 28 in total.

For the “negative” class, both approved and experimental drugs from DrugBank were selected. The antiviral drugs designed to target specific viral protein targets (such as remdesivir, tenofovir and taribavirin) were designated as neutral (“0”) class and were excluded from the model calibration. This is because the primary objective here is to target SARS-CoV-2 host interactome networks rather than individual viral proteins.

All available compounds from DrugBank and FooDB which were not included in the “positive” and “negative” classes were not used at the model calibration and parameter optimisation stage. Six thousand five hundred ninety-three compounds formed the input “negative” class; however, depending upon the specific parametrisation settings, the final number of negative class compounds varied between 1181 and 4260 due to the drugs with no connections being automatically removed.

## Supplementary Information

**Additional file 1.** Enriched pathways in SARS-CoV-2.

**Additional file 2.** Extended methods.

**Additional file 3.** Best parameters model with aggregated interactome.

**Additional file 4.** Best parameters model with wiki pathways.

**Additional file 5.** Anti-SARS-CoV-2 predicted drugs and foods.

**Additional file 6.** GSEA of anti-SARS-CoV-2 predicted foods.

**Additional file 7.** Manually curated anti SARS-CoV-2 compounds.

**Additional file 8.** Foods and compound concentrations.

## Data Availability

Supporting data and software can be found in https://bitbucket.org/iAnalytica/corona-ai-correlator/src/master/.
