# Supplementary material for: Network machine learning maps phytochemically rich “Hyperfoods” to fight COVID-19
Source: Hum Genomics. 2021 Jan 2;15:1. doi: 10.1186/s40246-020-00297-x (PMC7775839; doi:10.1186/s40246-020-00297-x)
Supplement: Supplementary file 2 — Additional file 2. Extended methods. [file 40246_2020_297_MOESM2_ESM.pdf]

Additional File 2 - Extended methods

1 Random walk with restarts

In brief, random walk with restarts models the probability of transition from the starting node in the graph to another linked node based on the network connectivity and the edge weights. By default, the graph was treated as undirected and all edges had a weight of 1.0. Then outgoing connection weights were normalized to the sum of weights to give the total probability of transition from the node of 1.0 resulting in the transition probability matrix $\boldsymbol{W}$. Parameter c (0.0-1.0) controls the probability of "jumping" back to the original node. *I.e., c* = 0.0 means unlimited wandering through the network resulting in a far-reaching propagation of the initial perturbation and a very smooth profile, while c = 1.0 means a hard restart to the initial position thus resulting in no signal being propagated beyond the starting nodes (i.e. the nodes directly affected by the drug or the disease). Random walk is an iterative algorithm and for each step the new perturbation profile $\mathbf{p}_i$ is given by the following equation:

$$\mathbf{p}_i = \mathbf{p}_{i-1} * \boldsymbol{W} * (1.0 - c) + c * \mathbf{p}_0,$$

where $\mathbf{p}_0$ is the starting profile, $\mathbf{p}_{i-1}$ - previous iteration profile and $\boldsymbol{W}$ is the transition probability matrix. Propagation algorithm was iterated until convergence when $|\mathbf{p}_i - \mathbf{p}_{i-1}|$ is less than a set tolerance value for each element.

2 Pearson correlation coefficient

The Pearson correlation coefficient is defined as:

$$\boldsymbol{\rho}_{j,covid} = \frac{cov(\boldsymbol{p}_j, \boldsymbol{p}_{covid})}{\sigma_j \sigma_{covid}}$$

Where $cov(\cdot)$ is covariance between molecule $\boldsymbol{p}_j$ and SARS-CoV-2 $\boldsymbol{p}_{covid}$ perturbation profiles, σ_j and σ_{covid} are the standard deviations of perturbation profiles $\boldsymbol{p}_j$ of molecule j and $\boldsymbol{p}^{covid}$ perturbation profile of SARS-CoV-2.

3. Parameter optimization, accuracy estimation and results aggregation

Pearson correlation coefficients between each drug and disease propagated profiles were calculated for the drugs/food molecules and for coronavirus affected gene sets. Ranges of parameters such as restart probability c, drug-gene connection thresholds and gene-gene interaction thresholds were explored to find the optimal parameters for drug/food molecule ranking.

Gene-gene connection threshold changes the number of connections in the interactome by filtering interactions based on their evidence levels. The higher the threshold is, the stricter the knowledge about the included interactions are, but at the cost of losing less studied interactions. The virus-gene and drug-gene thresholds control which “entry” points are included for the virus and drug effects on the interactome. Similarly, they threshold less studied virus-gene and drug-gene

interactions. The network propagation c parameter dictates how far the initial effect of the virus or the drug propagates through the entire interactome.

The following parameter ranges have been used: 1) Propagation parameter *c* for compounds: 0.0001, 0.0002, 0.0004, 0.0007, 0.0009, 0.001, 0.005, 0.01, 0.02, 0.04, 0.07, 0.1, 0.2, 0.4, 0.7; 2) Propagation parameter *c* for coronavirus-host profiles - 0.0001, 0.0002, 0.0004, 0.0007, 0.0009, 0.001, 0.005, 0.01, 0.02, 0.04, 0.07, 0.1, 0.2, 0.4, 0.7, 1.0; 3) STITCH (drug-gene, food-gene) confidence threshold/ minimum_connections/maximum_top_connections: 200/10/9999, 400/10/9999, 500/10/9999, 600/5/9999, 700/5/9999, 0/10/15, 0/10/25, 0/10/35, 0/10/50, 0/10/100, 0/10/200, 0/10/300; 4) STRING gene-gene confidence threshold: 0, 100, 150, 200, 250, 300, 400, 450, 500, 550, 600, 650, 700, 750; 5) Compound "positive" class target groups: "Target_Cell", "Target_Cell_Strict", "Target_Cell_Sympt", "Target_Cell_Sympt_Strict" 6) Target host gene sets for SARS-CoV-2: "score_wiki", "score20", "score25", "score30", "score40", "score_5_weighted", "entry_only".

The best parameters were established through cross-validation in 5 repeats of 5-fold stratified *k*-fold splitting for each parameter combination. Drugs were ranked by their profile correlations with the disease profile. Class separation threshold was set as the one resulting in the minimal difference between sensitivity and specificity. Balanced accuracy was used for establishing the best parameter combinations due to high class imbalance.

Median ranked (*r*-values) and their MADs were calculated for compounds independently for “Aggregated” and “WikiPathway” SARS-CoV-2 host interactomes. The ensemble of parameter settings in the range of balanced classification accuracies of 80-84.9% was used to provide consensus ranking of drug and food molecule candidates. The final ranking list for the two parameter sets was calculated using geometric mean of the *r*-values and MADs to guarantee that only the candidates scored highly using both sources of SARS-CoV-2 target genes would be at the top of the list. *r*-values were calculated for each compound as the sum of compounds in the “negative” class with the correlation coefficient higher than that of a given compound divided by the total number of the “negative” class compounds.

The toxic compounds were removed from the presented lists using literature and T3DB. For food molecules, we have also excluded compounds which are present in trace amounts (e.g. minerals) and/or are of non-natural origin.

4.GSEA pathway analysis

Pathway analytics was performed using gene set enrichment analysis (GSEA) via the Python GSEAPY package [1]. We used the random walk propagation algorithm on the initial SARS-CoV-2 host interactome to simulate the effects of SARS-CoV-2 on human interactome networks. This simulated genomic profile was used as input for the PreRank module of GSEA to find statistically significant enriched pathways/gene sets. KEGG v7.2 and Reactome v74 were used as default gene sets.

As a means of validating food predictions, we built genomic perturbation profiles of the predicted foods with anti-COVID-19 properties and subjected them to gene set enrichment analysis. Food genomic perturbation profiles were built by aggregating genomic perturbation profiles of their constituent molecules which represent the interactions between food molecules and the human interactome. Profiles of food molecules were weighted by their concentration in each of the food items (which can be found in the Additional file 8) and added together. The final food perturbation profiles were multiplied by the SARS-CoV-2 simulated genomic perturbation profile before being used as inputs to the PreRank module of GSEA. The statistical significance of the pathway overlap between each of the anti-COVID-19 predicted foods and SARS-CoV-2 was measured using Fisher exact test (Additional file 6). A threshold of FDR=0.05 was used to define the statistically significant enriched pathways in both groups.

5. Food map construction

The final food selection was based on the highest number and, where available, quantity of the anti-SARS-CoV-2 food compounds and is provided in the Additional file 8. Concentration of compounds within foods were extracted from the USDA Special Interest Database on Flavonoids [2].

An enrichment score for each food item was calculated as a weighted sum of the number of different molecules with anti-COVID-19 properties (phytochemical "diversity") and their relative abundance where the experimental concentration

data of molecules was available across all foods studied here. The enrichment score is defined as:

$$ES = \sum_i \log\left(\frac{\max(\boldsymbol{c}_i)}{st(\boldsymbol{c}_i)} + 1\right) + b_i$$

Where $\boldsymbol{c}_i$ is a vector with the concentrations of food molecule i across several samples of the food of interest, $st(\cdot)$ denotes standard deviation, and b_i is a binary indicator of the molecule-food association.

REFERENCES

1. Subramanian, A., et al., Gene set enrichment analysis: a knowledge-based approach for interpreting genome-wide expression profiles. Proc Natl Acad Sci U S A, 2005. 102(43): p. 15545-50.

2. Bhagwat S., et al., USDA's Expanded Flavonoid Database for the assessment of Dietary Intakes. U.S. Department of Agriculture, Agricultural Research Service, 2014. Nutrient Data Laboratory Home Page: http://www.ars.usda.gov/nutrientdata
